# Anterior Segment Optical Coherence Tomography (AS-OCT) 3D Observation of PreserFlo MicroShunt

**DOI:** 10.7759/cureus.72511

**Published:** 2024-10-27

**Authors:** Masaki Tanito, Tetsuro Omura, Mizuki Iida, Kana Murakami, Chisako Ida, Hinako Otani, Keigo Takagi, Akiko Harano, Sho Ichioka, Kazunobu Sugihara

**Affiliations:** 1 Department of Ophthalmology, Shimane University Faculty of Medicine, Izumo, JPN

**Keywords:** anterior segment optical coherence tomography (as-oct), filtration surgery, glaucoma surgery, minimally invasive glaucoma surgery (migs), preserflo microshunt

## Abstract

A 74-year-old Japanese woman with a history of primary open-angle glaucoma (POAG) in both eyes underwent PreserFlo MicroShunt (PFM) (Osaka, Japan: Santen Pharmaceutical, Co., Ltd.) implantation two years prior. Despite slit-lamp examination and standard anterior segment optical coherence tomography (AS-OCT) evaluations, the detailed condition of the stents was unclear. By using raster scanning and three-dimensional (3D) AS-OCT imaging, stent deformation and displacement were clearly identified, including a C-shaped deformation in the right eye. This type of deformation has rarely been reported and may result from compression by surrounding scar tissue. AS-OCT is widely known to be useful in perioperative evaluation of PFM, but our experience suggests that the addition of 3D imaging significantly enhances its utility in assessing the precise condition of the stent postoperatively. In this case, we were able to gain valuable insights into the stent placement, which may aid in future perioperative management of PFM complications.

## Introduction

The PreserFlo MicroShunt (PFM) (Osaka, Japan: Santen Pharmaceutical, Co., Ltd.) is a glaucoma surgical device used in filtration surgery, designed to reduce intraocular pressure (IOP) by draining aqueous humor from the anterior chamber into the subconjunctival space [1,2]. PFM offers the advantage of a simpler surgical procedure, as it does not require the creation of a scleral flap or iridectomy [1,2].

Anterior segment optical coherence tomography (AS-OCT) uses a light source with a wavelength of 1310 nm, which is longer than that used in posterior segment OCT, and employs a swept-source mechanism, allowing for deeper tissue penetration. It is used for the diagnosis and preoperative or postoperative evaluation of cataract, corneal and conjunctival diseases, and glaucoma. In the context of PFM surgery, AS-OCT has been used to evaluate bleb morphology [3-6], the angle and the length of device insertion [7-9], device obstruction [10], device exposure [11,12], and device displacement [13]. However, most of these reports are based on two-dimensional cross-sectional imaging, with few cases utilizing three-dimensional imaging [13]. Here, we report a case in which three-dimensional AS-OCT imaging was useful in evaluating the stent condition in a patient who underwent bilateral PFM implantation two years prior.

## Case presentation

A 74-year-old Japanese woman visited the Shimane University Hospital for evaluation and treatment of primary open-angle glaucoma in both eyes. She had a systemic medical history of untreated hypertension but no other significant medical issues. Four years ago, she underwent bilateral Tanito Microhook trabeculotomy combined with small-incisional cataract surgery. Two years ago, she underwent bilateral PFM implantation. Needling has not been performed, and there is no history of trauma. Currently, she is using a combination of tafluprost-timolol eye drops and brinzolamide-brimonidine eye drops in both eyes. IOP before the PFM surgery was approximately 18-21 mmHg in both eyes.

Upon examination, her best-corrected visual acuity (BCVA) was 0.6 in the right eye and 0.3 in the left eye, with both eyes showing -0.5D of myopia. Goldman IOP was 15 mmHg in the right eye and 18 mmHg in the left eye. The vertical cup-to-disc ratios were 0.8 in the right eye and 0.9 in the left eye, with glaucomatous rim thinning and nerve fiber layer defect observed in both eyes. Fundus OCT (RS-3000 Advance 2; Gamagori, Japan: Nidek Co., Ltd.) revealed significant thinning of the papillomacular nerve fibers in both eyes. Humphrey visual field testing (Dublin, CA: Carl Zeiss Meditec) showed a mean deviation of -6.58 dB in the right eye and -14.47 dB in the left eye with the central 30-2 program. In the central 10-2 program, the mean deviation was -8.64 dB in the right eye and -25.21 dB in the left eye. Foveal sensitivity was reduced to 31 dB in the right eye and 26 dB in the left eye, consistent with a glaucoma-related decrease in BCVA. Central corneal thickness measured by specular microscopy (EM-3000; Nagoya, Japan: Tomey Corp.) was 540 µm in the right eye and 549 µm in the left eye, with endothelial cell densities of 3118 cells/mm² in the right eye and 2684 cells/mm² in the left eye. Anterior chamber flare measured by flare meter (FM-600; Nagoya, Japan: Kowa Co. Ltd.) was 14.1 photon counts/ms in both eyes.

**Figure 1:**
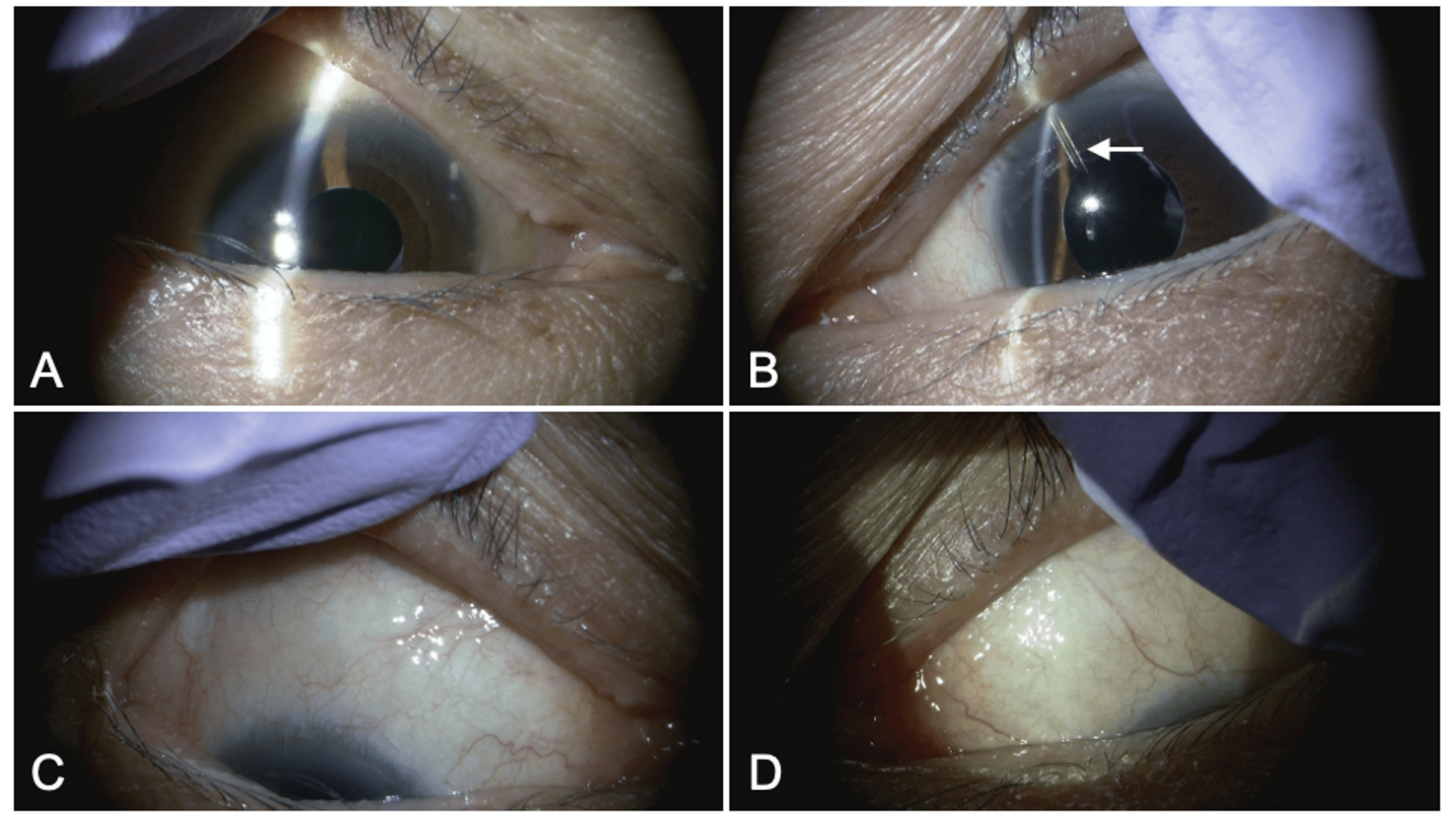
Slit lamp findings of the right eye (A and C) and left eye (B and D). (A and B) In frontal view, the stent tip (arrow) is observed in the anterior chamber of the left eye, but not in the right eye. (C and D) In downward gaze, due to conjunctival thickening, the precise location and condition of the stent are difficult to observe in downward gaze due to conjunctival thickening.

Slit-lamp examination showed clear corneas and deep anterior chambers in both eyes, with no signs of inflammation (Figures 1A-1D). The tip of the PFM in the left eye was observed in the nasal-superior quadrant of the anterior chamber, but the stent tip was not visible in the right eye (Figure 1B). No bleb formation was observed in either eye, and the conjunctiva in the nasal-superior quadrant was thickened, making it difficult to visualize the stent in detail (Figures 1C, 1D). The intraocular lenses were well-positioned and showed no signs of posterior capsular opacification.

**Figure 2:**
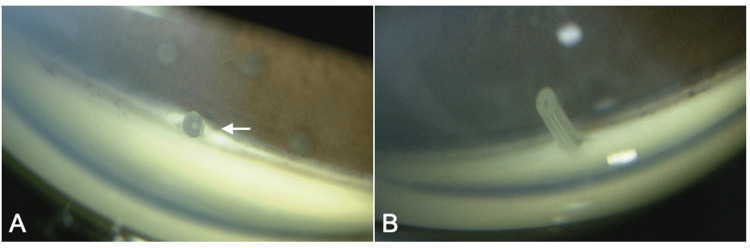
Gonioscopic findings of the right eye (A) and left eye (B). The stent tip is visible in the angle of both eyes, but the insertion length of the right eye (arrow) is shorter than the left eye.

Gonioscopy revealed Shaffer grade 4 open angles in both eyes, with mild pigmentation (Figures 2A, 2B). There was no peripheral anterior synechiae, and the trabeculotomy clefts were not clearly visible in either eye. In both eyes, the stent tips were visible at the nasal-superior angle, but the insertion length of the stent in the right eye (Figure 2A) was shorter than that in the left eye (Figure 2B).

**Figure 3:**
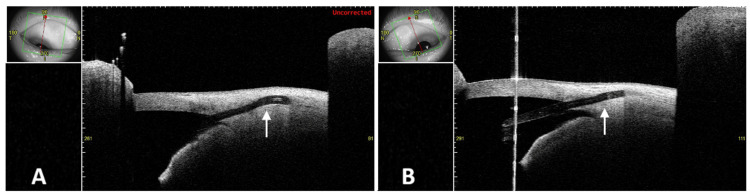
Conjunctival cross-sectional observation using AS-OCT 2D mode of the right eye (A) and left eye (B). The stent (arrow) beneath the conjunctiva is visible in both eyes, but it is difficult to determine the overall trajectory and posterior position of the stent. No distinct bleb formation is observed in either eye. The small image in the upper left corner of each panel indicates the scan direction, as well as the length and width of the raster scan. AS-OCT: anterior segment optical coherence tomography

To evaluate subconjunctival condition of the stents, AS-OCT (CASIA2 Advance; Nagoya, Japan: Tomey Corp.) was performed. The software version used was 50.6A.02, and the bleb observation mode was applied to scan the nasal-superior corneal limbus perpendicularly. The raster scans were performed with the default settings of the device software (12 mm length, 400 pixels/line B-scan × 12 mm width, 256 lines with no slice repeat). In the perpendicular 2D mode, the stents were visible beneath the thickened conjunctiva in both eyes, but it was difficult to assess the full trajectory of the stents (Figures 3A, 3B). No significant bleb formation was observed in either eye.

**Figure 4:**
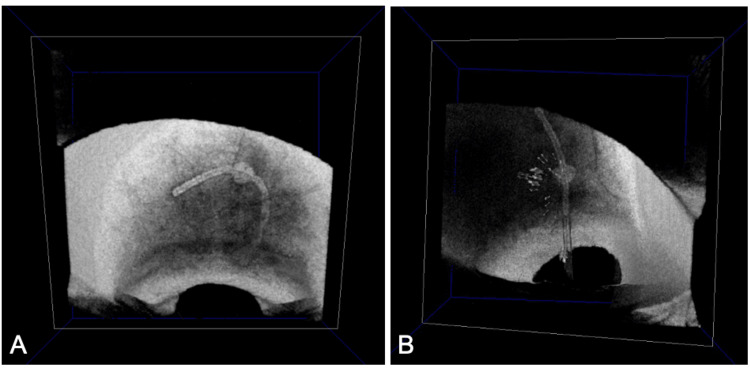
Ocular surface observation using AS-OCT 3D mode of the right eye (A) and left eye (B). (A) In the right eye, the stent is observed to be curved in a C shape, suggesting that the fin may have dislodged from the scleral pocket, resulting in displacement. (B) In the left eye, the stent’s position is appropriate, though the stent behind the fin is slightly curved, as clearly observed. AS-OCT: anterior segment optical coherence tomography

Using the software’s viewer, the vertical and horizontal axes were adjusted to display the entire stent in the C-scan window. Upon performing 3D imaging, the positioning of the stents beneath the conjunctiva was visualized (Figures 4A, 4B and Videos 1, 2). The stent in the right eye was found to be curved in a C-shape, and the fin appeared to have dislodged from its original position (Figure 4A and Video 1). The left eye showed no significant displacement, although the posterior half of the stent was slightly curved (Figure 4B and Video 2). As the patient was already on full medication, we decided to monitor IOP and visual fields and consider bleb revision or device realignment if necessary.

**Video 1:** AS-OCT 3D observation of the right eye. AS-OCT: anterior segment optical coherence tomography; RE: right eye

**Video 2:** AS-OCT 3D observation of the left eye. AS-OCT: anterior segment optical coherence tomography; LE: left eye

## Discussion

We encountered a case where it was difficult to fully assess the condition of the stents using slit-lamp examination and standard AS-OCT cross-sectional imaging. In this case, raster scanning and 3D observation clearly revealed the deformation and displacement of the stents. Common complications (>5%) after PFM implantation include choroidal detachment and shallow anterior chamber [14]. Relatively rare complications (<5%) include bleb encapsulation, iridocorneal touch, blocked lumen, and corneal edema, while very rare complications (<1%) include tube migration and device erosion [14]. Of all these complications, AS-OCT is useful for evaluating the underlying conditions, assessing the severity, and monitoring the progression. While the utility of AS-OCT in the perioperative management of PFM is well recognized, based on our experience, we believe that its usefulness is further enhanced when combined with 3D visualization.

The C-shaped deformation of the PFM observed in our case has not been reported in the literature, except in our previous case report [13]. This type of deformation may result from compression by surrounding scar tissue. It is recommended during PFM surgery to ensure that the Tenon’s capsule does not impinge on the device during insertion [2]. Factors such as Tenon’s capsule entrapment, posterior tube elevation, and bleb fibrosis may contribute to such stent displacement and deformation.

## Conclusions

We encountered a case where the three-dimensional imaging provided by AS-OCT enabled detailed observation of stent placement. In post-PFM eyes, AS-OCT with 3D imaging, in addition to 2D imaging, proved to be particularly useful.
